# Commentary: Musculoskeletal adverse events in dogs receiving bedinvetmab (Librela)

**DOI:** 10.3389/fvets.2025.1628681

**Published:** 2025-07-16

**Authors:** Matthew Warren Brunke, Curtis Wells Dewey

**Affiliations:** ^1^Veterinary Referral Associates, Gaithersburg, MD, United States; ^2^Elemental Pet Vets PLLC, Freeville, NY, United States

**Keywords:** Librela, bedinvetmab, osteoarthritis, adverse (side) effects, musculoskeletal disorders, pharmacovigilance

We are writing in response to the article “*Musculoskeletal adverse events in dogs receiving bedinvetmab (Librela)”* by Farrell et al. (1), which presents an important and timely evaluation of the safety profile of bedinvetmab using data from the European Medicines Agency's EudraVigilance database.

The authors' specialist-led disproportionality analysis reveals a significantly elevated rate of serious musculoskeletal adverse events (MSAEs)—including ligament and tendon injuries, polyarthritis, fractures, musculoskeletal neoplasia, and septic arthritis—in dogs treated with bedinvetmab compared to six other osteoarthritis medications. The study also reports expert consensus on a strong suspicion of a causal association between bedinvetmab and accelerated joint destruction. These findings align with prior FDA pharmacovigilance concerns over musculoskeletal and neurological events following Librela use.

While bedinvetmab represents an innovative approach to canine osteoarthritis management through NGF inhibition, the potential for rapid joint degradation and other serious events warrants significant caution. The parallel with adverse outcomes seen in human anti-NGF trials—particularly rapidly progressive osteoarthritis—should not be overlooked.

What is particularly concerning is the study's finding that the final case narratives and diagnoses in the EudraVigilance database often differed from those originally reported by practicing veterinarians, due to modifications made by the marketing authorization holder, Zoetis. This undermines the reliability of the database and may hinder accurate signal detection and pharmacovigilance. More specifically, if the terminology for a potential adverse drug event does not exist among the options currently available in the EudraVigilance database, one will be assigned from the list of options provided by Zoetis.

This raises urgent questions about how veterinarians can ensure that their reports are accurately represented in regulatory data. It would be highly valuable for your journal—or a regulatory agency—to provide clear guidance on the following points:

1. How veterinary professionals can submit adverse event reports directly to competent national authorities or the EMA to ensure transparency.
2. Whether they can request verification of how their reports are represented in EudraVigilance.
3. Best practices for documenting musculoskeletal and neurological adverse events (AEs) using standardized, unambiguous terminology.
4. How to follow up when discrepancies in submitted reports are suspected.

Veterinarians need assurance that their clinical observations are faithfully recorded and reflected in pharmacovigilance systems. Without this, both animal safety and regulatory accountability are compromised.

We share the view that ongoing reporting and open dialogue regarding adverse events (AEs) associated with therapies such as bedinvetmab are essential for safeguarding companion animal welfare, promoting translational research, and upholding clinical standards in veterinary medicine.

1. Standardized Terminology for Adverse Events (AEs): One of the primary challenges identified in the current literature is the inconsistent terminology used to describe musculoskeletal AEs. To support improved data clarity and comparability, we recommend that clinicians refer to existing frameworks, including the following:

- The FDA Center for Veterinary Medicine (CVM) Reporting Standards: In the U.S., adverse events can be submitted using Form FDA 1932a, which captures clinical observations and product-related issues. Although a formal dictionary such as MedDRA is not in routine veterinary use, consistent application of clear and clinically descriptive terms (e.g., “lameness,” “joint pain,” “muscle atrophy”) can significantly improve data utility.
- Veterinary Dictionary for Drug Regulatory Activities (VeDDRA): VeDDRA, developed by the EMA, offers standardized veterinary-specific terminology that is especially useful for categorizing musculoskeletal and neurologic events. Wider adoption of this or a similar system in North America could enhance pharmacovigilance consistency.
- Severity Grading: AE grading systems such as the VCOG-CTCAE, although originally designed for oncology, can be adapted to classify adverse event severity in musculoskeletal cases, improving both clinical documentation and regulatory reporting.

2. Next Steps for Clinicians in Reporting AEs:

- Thorough Clinical Documentation: Clinicians should record baseline physical and orthopedic examinations, treatment timing, onset and duration of signs, diagnostic imaging findings, and the clinical course of recovery or progression. Videos or images can also be helpful for conveying nuanced findings such as subtle lameness or postural abnormalities.
- Reporting Routes:

Adverse events may be submitted directly to the following:

° The FDA CVM using Form FDA 1932a, which allows voluntary submission of suspected AEs.
° The drug manufacturer, which is a more commonly used route due to ease of access.

- Access Barriers and Systemic Limitations:

One of the ongoing challenges in AE reporting is that primary care veterinarians and pet owners often lack practical and easy access to these formal reporting systems. The FDA reporting form is not user-friendly, requires considerable time, and is underutilized by general practitioners due to workload and awareness limitations.

As a result, AEs are more frequently reported directly to the pharmaceutical manufacturer, which is convenient but introduces inherent bias. There is a valid concern that manufacturers may utilize vague or overly broad terminology when coding AEs, which may lead to underreporting or reclassification of events in a way that reduces their visibility as safety signals. For example, labeling a post-injection gait abnormality simply as “age-related decline” or “orthopedic flare” may diminish its relevance in safety surveillance databases.

Therefore, while we encourage clinicians to report suspected AEs in any form possible, we strongly advocate for improved access to independent, transparent reporting pathways and encourage regulatory agencies and veterinary professional organizations to simplify and promote the use of direct-to-regulator systems (e.g., the FDA CVM).

3. Oversight and Human Medicine Comparison: In human medicine, AE reporting is standardized under systems such as MedWatch (FDA CDER) and utilizes terminology from MedDRA, which allows for consistent classification, severity grading, and statistical evaluation. Veterinary medicine lacks a comparable unified system, but international harmonization efforts—such as coordinated use of VeDDRA and transparent reporting infrastructure—could support similar advancements.

Additionally, we have attached as Supplementary material three documents. One is a reporting form for Librela-associated AEs in dogs, a similar form for Solensia (frunevetmab, a similar drug for cats), and a “How to Report Adverse Side Effects for Librela or Solensia” guide.

We commend Farrell et al. for bringing this issue to light and urge the broader veterinary community to advocate for greater transparency and accuracy in adverse event reporting.
