# Supplementary material for: Commentary: Musculoskeletal adverse events in dogs receiving bedinvetmab (Librela)
Source: Front Vet Sci. 2025 Jul 16;12:1628681. doi: 10.3389/fvets.2025.1628681 (PMC12307179; doi:10.3389/fvets.2025.1628681)
Supplement: Supplementary file 1 [file Data_Sheet_1.pdf]

HOW TO REPORT ADVERSE SIDE EFFECTS AFTER ADMINISTRATION OF LIBRELA OR SOLENSIA

As a result of concerns associated with possible adverse effects after administration of Librela or Solensia (https://www.frontiersin.org/journals/veterinary-science/articles/10.3389/fvets.2025.1581490/full and https://www.youtube.com/watch?v=ywu11n8KXWo , just a few examples providing good information), it is the responsibility of the community of veterinarians and / or pet owners to report ALL of those potential adverse effects. Zoetis and the relevant authorities in the specific countries are asking for this to be done. This is the best action to collectively assess the risks versus benefits associated with those medications.

To ensure that no translation errors occur as described in the most recent publication (see link above), **it is best to report to both, the manufacturer Zoetis directly as well as to the relevant authority in the specific country.** During that process, proper tracking of these reports has to be ensured. Therefore, you will need a tracking number. You can only get this when calling or emailing. Using the online form does NOT provide a tracking number! Please find the necessary information and links on the next two pages.

HOW TO REPORT DIRECTLY TO ZOETIS:

To report a possible adverse event associated with a Zoetis product, including human exposure, contact the Veterinary Medical Information Support Team in your region.
Use this link: Contact Zoetis Animal Health | Zoetis

**** ENSURE TO CALL OR EMAIL ** ONLY THAT WILL PROVIDE A REFERENCE NUMBER THAT CAN BE TRACT ** DONT USE THE ONLINE FORM: NO REFERENCE # IS GENERATED**

To report in the UK but email works in the EU too:
Call Zoetis;0345 300 8034. Report the event and ask for a reference number. Track the development of your submission using that reference number.
or email Zoetis: customersupportUK@zoetis.com Report the event and ask for a reference number. Track the development of your submission using that reference number.

To report in the US:
Call 1-888-Zoetis1 (1-888-963-8471). Report the event and ask for a reference number. Track the development of your submission using that reference number

For other international regions, this Link: Contact Zoetis Animal Health | Zoetis provides contact information for the regions as shown below. Select your region and country and call the phone number provided for your country. Report the event and ask for a reference number. Track the development of your submission using that reference number:

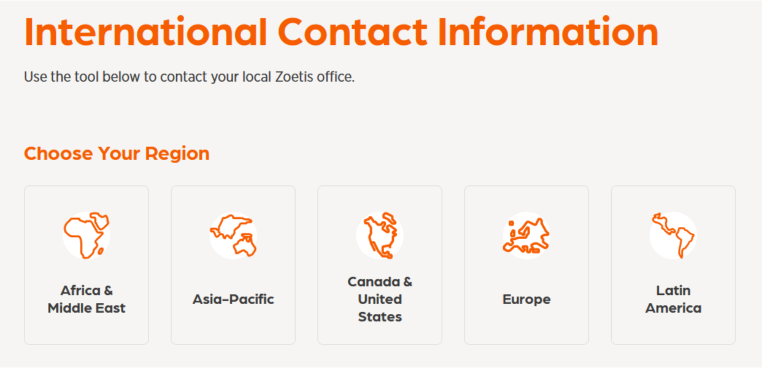

HOW TO SIMULTANEOUS REPORT TO THE TO THE RELEVANT AUTHORITY IN THE SPECIFIC COUNTRY:

In the UK:
adverse.events@vmd.gov.uk

In Europe:
Heads of Medicines Agencies: National Contacts

In the USA:
https://www.fda.gov/safety/medwatch-fda-safety-information-and-adverse-event-reporting-program
https://www.ncbi.nlm.nih.gov/books/NBK208615/

In Canada:
https://www.canada.ca/en/health-canada/services/drugs-health-products/medeffect-canada/adverse-reaction-reporting.html

In Australia:
https://www.tga.gov.au/safety/reporting-problems

For any other country, check and see which agency to report to.

Please share this with all veterinarians and orthopedic colleagues – everybody must be aware. It is appreciated that this takes time, but it is really important!
