# Supplementary material for: Commentary: Musculoskeletal adverse events in dogs receiving bedinvetmab (Librela)
Source: Front Vet Sci. 2025 Jul 16;12:1628681. doi: 10.3389/fvets.2025.1628681 (PMC12307179; doi:10.3389/fvets.2025.1628681)
Supplement: Supplementary file 2 [file Data_Sheet_2.pdf]

Librela Adverse Event Report Submission Form

Reporting veterinarian:

Qualification:

Animal name:

Owner name:

Dog age: years months

Sex: ☐ Male ☐ Female ☐ Neutered

Breed:

Weight: ☐ kg ☐ lb

Product name:

- ☐ Librela 5mg solution for injection for dogs
- ☐ Librela 10mg solution for injection for dogs
- ☐ Librela 15mg solution for injection for dogs
- ☐ Librela 20mg solution for injection for dogs
- ☐ Librela 30mg solution for injection for dogs

Reason for using the product: Mild OA ☐ Severe OA ☐ Other:

Start date (day/month/year):

Last dose (day/month/year):

Number of doses:

Description of adverse event:

Requested term for submission to EudraVigilance (if known):

Do I think the product contributed to the adverse signs observed? ☐ Yes ☐ No

Number of animals treated*:

Number of animals reacted:

Number of animals died or killed:

Time to onset:

- [ ] **Immediate:** Minutes to hours
- [ ] **Early:** Hours to 3-days
- [ ] **Intermediate:** Days to 4-weeks
- [ ] **Late:** Over 4-weeks

Is the reaction serious?

- [ ] **Serious:** My patient was at substantial risk of death at the time of the event, or the event itself posed an immediate threat to my patient's life
- [ ] **Serious:** The adverse event necessitated a prolonged hospital stay
- [ ] **Serious:** The adverse event caused a substantial disruption of my patient's ability to conduct normal life functions or resulted in a significant, lasting, or permanent change, impairment, or damage to body function/structure, physical activities, and/or quality of life.
- [ ] **Not serious:** None of the above

Outcome:

- [ ] **Recovered/resolved:** Full recovery from the adverse event. Clinical signs are absent
- [ ] **Recovered/resolved with sequelae:** Incomplete recovery with ongoing effects, permanent damage, or disability as a result of the adverse event
- [ ] **Ongoing**
- [ ] **Fatal**
- [ ] **Unknown**

Concurrent medication:

***Note:** Veterinarians aren't expected know the total number of dogs they've treated with Librela across their entire practice or over a long period. Asking for this broad cumulative number for every adverse event report would be unrealistic and burdensome. Consequently, for Librela, where you are reporting an adverse event for a single dog that received the drug, the number of animals treated is listed as "1".
