# Supplementary material for: Commentary: Musculoskeletal adverse events in dogs receiving bedinvetmab (Librela)
Source: Front Vet Sci. 2025 Jul 16;12:1628681. doi: 10.3389/fvets.2025.1628681 (PMC12307179; doi:10.3389/fvets.2025.1628681)
Supplement: Supplementary file 3 [file Data_Sheet_3.pdf]

Solensia Adverse Event Report Submission Form

Reporting veterinarian: ☐

Qualification: ☐

Animal name: ☐

Owner name: ☐

Cat age: ☐ years ☐ months

Sex: ☐ Male ☐ Female ☐ Neutered

Breed: ☐

Weight: ☐ ☐ kg ☐ lb

Product name:

☐ Solensia 7mg/ml solution for injection for cats

Reason for using the product: Mild OA ☐ Severe OA ☐ Other: ☐

Start date (day/month/year): ☐

Last dose (day/month/year): ☐

Number of doses: ☐

Description of adverse event:

☐

Requested term for submission to EudraVigilance (if known): ☐

Do I think the product contributed to the adverse signs observed? ☐ Yes ☐ No

No. of cats treated* ☐ **No.of cats reacted** ☐ **No. of cats died/killed** ☐

Time to onset:

☐ **Immediate:** Minutes to hours

☐ **Early:** Hours to 3-days

☐ **Intermediate:** Days to 4-weeks

☐ **Late:** Over 4-weeks

Is the reaction serious?

- ☐ **Serious:** My patient was at substantial risk of death at the time of the event, or the event itself posed an immediate threat to my patient's life
- ☐ **Serious:** The adverse event necessitated a prolonged hospital stay
- ☐ **Serious:** The adverse event caused a substantial disruption of my patient's ability to conduct normal life functions or resulted in a significant, lasting, or permanent change, impairment, or damage to body function/structure, physical activities, and/or quality of life.
- ☐ **Not serious:** None of the above

Outcome:

- ☐ **Recovered/resolved:** Full recovery from the adverse event. Clinical signs are absent
- ☐ **Recovered/resolved with sequelae:** Incomplete recovery with ongoing effects, permanent damage, or disability as a result of the adverse event
- ☐ **Ongoing**
- ☐ **Fatal**
- ☐ **Unknown**

Concurrent medication:

***Note:** Veterinarians aren't expected know the total number of cats they've treated with Solensia across their entire practice or over a long period. Asking for this broad cumulative number for every adverse event report would be unrealistic and burdensome. Consequently, for Solensia, where you are reporting an adverse event for a single cat that received the drug, the number of animals treated is listed as "1".
